# Therapeutic effect of percutaneous kyphoplasty combined with anti-osteoporosis drug on postmenopausal women with osteoporotic vertebral compression fracture and analysis of postoperative bone cement leakage risk factors: a retrospective cohort study

**DOI:** 10.1186/s13018-019-1499-9

**Published:** 2019-12-18

**Authors:** Shuaihao Huang, Xiaowen Zhu, Dan Xiao, Jianxiong Zhuang, Guoyan Liang, Changxiang Liang, Xiaoqing Zheng, Yuhong Ke, Yunbing Chang

**Affiliations:** 1 Department of Orthopedics, Guangdong Provincial People’s Hospital, Guangdong Academy of Medical Sciences, Guangzhou, 510080 Guangdong Province China; 2 Department of Gynaecology and Obstetrics, Guangdong Provincial People’s Hospital, Guangdong Academy of Medical Sciences, Guangzhou, 510080 Guangdong Province China

**Keywords:** Percutaneous kyphoplasty, Osteoporosis, Bone cement leakage, Risk factor

## Abstract

**Background:**

The purpose of this study is to explore the therapeutic effect of percutaneous kyphoplasty (PKP) combined with anti-osteoporosis drug, zoledronic acid, on postmenopausal women with osteoporotic vertebral compression fracture (OVCF) and to perform an analysis of postoperative bone cement leakage risk factors.

**Methods:**

A total of 112 OVCF patients, according to therapeutic regimens, were divided into control group (*n* = 52, treated with PKP) and observation group (*n* = 60, treated with PKP and zoledronic acid injection).

**Results:**

Postoperative tumor necrosis factor-α and interleukin-6 levels were significantly decreased in the two groups, compared with those before treatment (both *P* < 0.05); bone mineral density (BMD), serum bone gla protein (BGP), and vertebral height ratio of injured vertebrae were significantly increased, and procollagen type I N-terminal propeptide (PINP), Cobb angle, visual analogue scale/score (VAS), and Oswestry disability index (ODI) were significantly decreased compared with those before treatment (all *P* < 0.05). There were significantly higher changes in difference value of BMD, PINP, BGP, vertebral height ratio of injured vertebrae, Cobb angle, VAS, and ODI levels and significantly better therapeutic effect in the observation group than those in the control group (all *P* < 0.05). Multivariate logistic regression analysis showed that the use of zoledronic acid, vertebral height ratio of injured vertebrae, and ODI were independent factors affecting the therapeutic effect, and that the dosage of bone cement, and peripheral vertebrae wall damage were independent risk factors causing postoperative bone cement leakage. There were no significant differences in postoperative bone cement leakage rate between the two groups.

**Conclusions:**

Peripheral vertebrae wall damage and the dosage of bone cement are independent risk factors causing bone cement leakage in OVCF patients treated with PKP. PKP combined with zoledronic acid has an improvement effect on the condition of postmenopausal women with OVCF and reduces the inflammation and pain in patients, which is beneficial to clinical treatment.

## Background

With the gradual improvement of people's living standards, the aging population is growing. Statistics show that the incidence of osteoporosis has risen to the 7th in the world's common diseases [1–3]. A report reveals about 10 million people over 50 years old in the USA have osteoporosis, and another 33 million people have symptoms of low bone mass [4]. Osteoporosis is a systemic disease that is characterized by a decrease in bone mass and bone mechanical strength and an increase in bone fragility which can lead to an increased risk of fracture [5–8]. The clinical symptoms of osteoporosis are mostly multiple pains, humpback, and height reduction.

Patients with osteoporosis can suffer from fracture when subjected to low-energy shock due to the increased bone fragility, and the fractures are often found in the pelvis, vertebrae, hip, and proximal humerus, clinically known as osteoporotic vertebral compression fracture (OVCF) [9]. The incidence of OVCF in women is significantly higher than that in men, especially in postmenopausal women, which may be associated with changes in estrogen levels of postmenopausal women [10]. The clinical manifestations of OVCF are mainly unbearable pain and inability to walk, which severely affects the quality of life of patients, and some patients with OVCF have mental disorder and venous thrombosis [11]. Therefore, there is a need in the clinic to find a solution to resolve this problem.

Percutaneous kyphoplasty (PKP) and percutaneous vertebroplasty (PVP) have achieved some results in the treatment of OVCF. These two regimens characterized by small trauma, high safety, and good analgesic effect have been widely popularized in the clinic [12]. As a derivative therapeutic regimen of PVP, PKP not only optimizes PVP in terms of safety and analgesic effect, but also overcomes the shortcomings of PVP, effectively increasing the height of injured vertebrae [13]. Zoledronic acid is the most commonly used anti-osteoporosis drug in clinical practice, which reduces the risk of fracture by increasing the bone mineral density of the lumbar vertebrae [14]. At present, there are few Chinese articles about zoledronic acid combined with PKP for the treatment of OVCF in postmenopausal women.

Therefore, in this study we administrated zoledronic acid combined with PKP to postmenopausal women with OVCF to observe its clinical efficacy, providing a reference for clinicians.

## Methods

### Patients

A total of 112 OVCF patients at an average age of 51.1 ± 3.2 ranging from 45 years to 55 years who were treated in Guangdong Academy of Medical Sciences, Guangdong Provincial People's Hospital from January 2015 to June 2017 were collected. The compressed vertebrae of all patients showed high signal on MRI-T2 weighted image, and X-ray examination indicated the presence of OVCF. This study was approved by the Medical Ethics Committee of Guangdong Academy of Medical Sciences, Guangdong Provincial People's Hospital, and subjects gave informed consent to the work.

### Inclusive and exclusive criteria

Inclusive criteria include the following: postmenopausal women, the site of compressed vertebrae with low back pain, osteoporosis indicated by pathological examination, complete clinical data, and PKP performed for the first time.

Exclusive criteria include the following: patients with leukemia, hemophilia, immune disease, and idiopathic thrombocytopenia; patients complicated with vertebral tumors; patients with nerve root dysfunction; patients with mental disorder; a history of allergies to zoledronic acid; patients who received conservative treatment; and patients complicated with osteoporosis due to other related diseases such as spinal tuberculosis.

### Sources of drugs

Vitamin D2 Lin Pu Gai Pian (a compound preparation including calcium hydrophosphate, calcium gluconate, and vitamin D2) was purchased from the Beijing Huarun Double-Crane Pharmaceutical Co., Ltd., China; zoledronic acid was obtained from the Sinopharm Group Guorui Pharmaceutical Co., Ltd., Anhui, China.

### Therapeutic regimens

Patients were divided into control group (*n* = 52) and observation group (*n* = 60) according to their therapeutic regimens. All patients were treated with PKP. During the surgical procedure, the sites of the injured vertebrae and pedicle of the vertebral arch were determined under the guidance of a C-arm X-ray machine. The patient was injected with antibiotics 30 min before surgery. The patient was placed in a prone position, the abdomen suspended, anesthetized (general anesthesia) by tracheal intubation, sterilized, and the drapes spread. The needle insertion direction was adjusted according to the patient's vertebral compression condition, keeping the needle insertion path in the center of the compressed vertebrae. The puncture needle was inserted into the 1/3 site in front of the lesion of the vertebrae through the pedicle of the vertebral arch, and the puncture site was confirmed by a C-arm X-ray machine. Balloon dilatation was performed on both sides of the vertebrae after a successful puncture. Bone cement of 2.0–2.5 mL was injected to each side of the vertebrae according to patient's condition. After the errorless frontal and lateral film was confirmed by a C-arm X-ray machine, the T-shaped cannula was repeatedly rotated. The cannula was taken out after the bone cement was fixed (stirring for 15 min). The incision was sutured. During the postoperative management, all patients lay on the back after the surgery and were allowed to turn over 4 h later. Patients were allowed to move around gradually under the protection of thoracolumbosacral orthosis 3–5 days after the surgery. Patients in the observation group were administrated with vitamin D2 Lin Pu Gai Pian (2 tablets/time, 3 times/day, for 3 months) plus zoledronic acid (4 mg/time, intravenous drip of 100 mL diluted normal saline with the drip duration greater than 15 min, only administration once) on the 2^nd^ postoperative day; patients in the control group were treated with vitamin D2 Lin Pu Gai Pian only. The patient was reviewed by X-ray film 3 months after the treatment.

### Source of reagents

Interleukin-6 (IL-6), tumor necrosis factor-α (TNF-α), serum procollagen type I N-terminal propeptide (PINP), and serum bone gla protein (BGP) ELISA kits were obtained from the Shanghai Meilian Biotechnology Co., Ltd., China.

### Detection method

IL-6, TNF-α, PINP, and BGP were detected using ELISA kits, which were performed according to the manufacturer's instructions. Three duplicate microwells were set, and the experiment was repeated three times. Bone mineral density (BMD) of the injured vertebrae was measured by X-ray absorption method.

### Outcome measurements

For the main outcome measurements, the curative effect after treatment was observed and evaluated by the Common Diagnosis and Classification Methods in Orthopedics and Functional Outcome Scale which were issued by Liu [15]. Changes in IL-6, TNF-α, PINP, BGP, and BMD before treatment and 1 week after treatment were observed. Vertebral height ratio of injured vertebrae 3 months after surgery and the Cobb angle were calculated. Risk factors affecting clinical efficacy were analyzed by multivariate logistic regression analysis.

For the secondary outcome measurements, pain degree before and 1 week after treatment was evaluated using visual analogue scale/score (VAS) with a total score of 10; the higher the score, the more severe the pain. Dysfunction before and 1 week after treatment was evaluated using Oswestry disability index (ODI) with a total score of 50; the higher the score, the more severe the dysfunction. The condition of postoperative bone cement leakage was observed. Risk factors causing postoperative bone cement leakage were analyzed by multivariate logistic regression analysis.

### Statistical analysis

SPSS 20.0 software package (Guangzhou Pomine Info. Tech. Co., Ltd., China) was used to analyze the data; the data were drawn using GraphPad Prism 7 (Cabit Information Technology Co., Ltd., Shanghai, China). The normal distribution of data was detected by K–S test; the enumeration data were expressed as the rate (%); the chi-squared test was used and shown as *χ*^2^; the ranked data were analyzed using the rank sum test, and expressed as *Z*. The measurement data that conformed to normal distribution and homogeneity of variance were expressed as mean ± SD; comparison between two groups was performed using independent sample *t*-test; comparison between before and after treatment in a group was carried out by paired *t*-test, and expressed as *t*. The data that did not conform to normal distribution and homogeneity of variance were analyzed by non-parametric test, and expressed as *Z*. Risk factors causing postoperative bone cement leakage were analyzed by multivariate logistic regression analysis. There was a significant difference at *P* < 0.05.

## Results

### Clinical data

There were no significant differences in age, body mass index (BMI), marital status, past medical history, place of residence, degree of education, cause, site of injured vertebrae, and dosage of bone cement between the two groups (all *P* > 0.05, Table 1).

**Table 1:** Clinical data (*n*, %)

Term	Control group (*n* = 52)	Observation group (*n* = 60)	*t*/*χ*^2^/*Z*	*P*
Age (year)	51.60 ± 3.30	50.70 ± 3.20	1.510	0.134
BMI (kg/m^2^)	23.54 ± 1.84	22.98 ± 1.92	1.569	0.119
Marital status			0.033	0.984
Married	43 (82.69)	50 (83.33)		
Unmarried	3 (5.77)	3 (5.00)		
Divorced	6 (11.54)	7 (11.67)		
Past medical history				
Hypertension	15 (28.85)	21 (35.00)	0.484	0.487
Diabetes	13 (25.00)	19 (31.67)	0.607	0.436
Hyperlipidemia	12 (23.08)	18 (30.00)	0.681	0.409
Coronary heart disease	5 (9.62)	8 (13.33)	0.375	0.540
Place of residence			0.718	0.397
City	42 (80.77)	52 (86.67)		
Rural area	10 (19.23)	8 (13.33)		
Degree of education			1.754	0.185
≥ Senior high school	33 (63.46)	45 (75.00)		
< Senior high school	19 (36.54)	15 (25.00)		
Cause			0.296	0.863
Falling down	32 (61.54)	34 (56.67)		
Sprain	15 (28.85)	20 (33.33)		
Other	5 (9.61)	6 (10.00)		
Site of injured vertebrae			−0.432	0.666
T7	4 (6.45)	6 (7.32)		
T8	1 (1.61)	1 (1.22)		
T9	3 (4.84)	2 (2.44)		
T10	4 (6.45)	5 (6.10)		
T11	3 (4.84)	8 (9.76)		
T12	13 (20.97)	18 (21.95)		
L1	11 (17.74)	15 (18.29)		
L2	8 (12.90)	10 (12.20)		
L3	7 (11.29)	8 (9.76)		
L4	6 (9.68)	6 (7.32)		
L5	2 (3.23)	3 (3.66)		
Dosage of bone cement (mL)	4.34 ± 1.10	4.65 ± 1.30	1.361	0.180

*BMI* body mass index

### Changes in inflammatory factor, bone metabolism indexes, vertebral height, and VAS and ODI scores before and after treatment

**Fig. 1:**
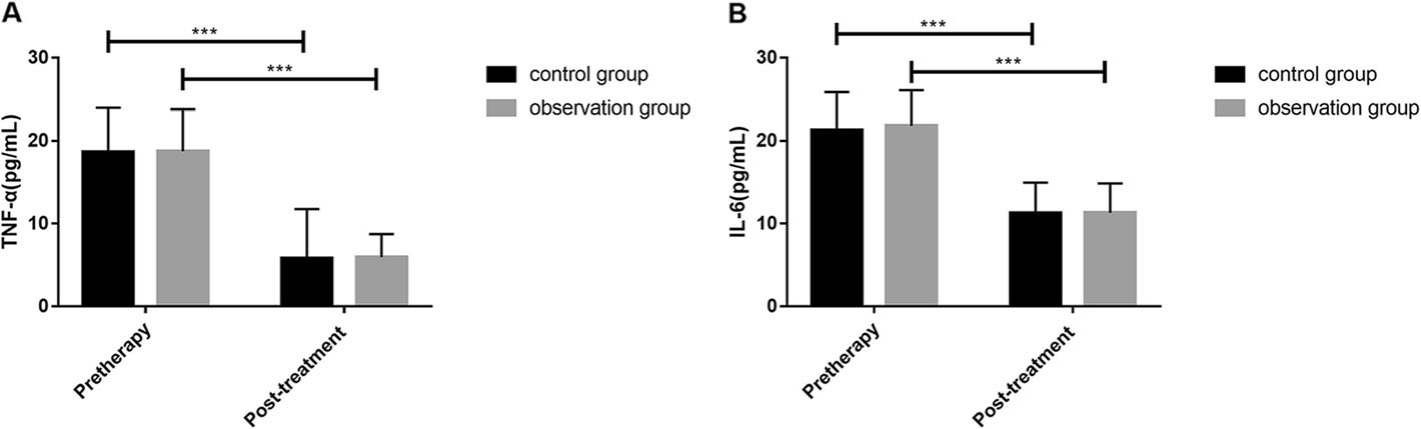
Pre and posttreatment TNF-α (**a**) and IL-6 (**b**) levels. ^***^There was a difference in a group before and after treatment (*P* < 0.001). TNF-α, tumor necrosis factor-α; IL-6, interleukin-6

**Fig. 2:**
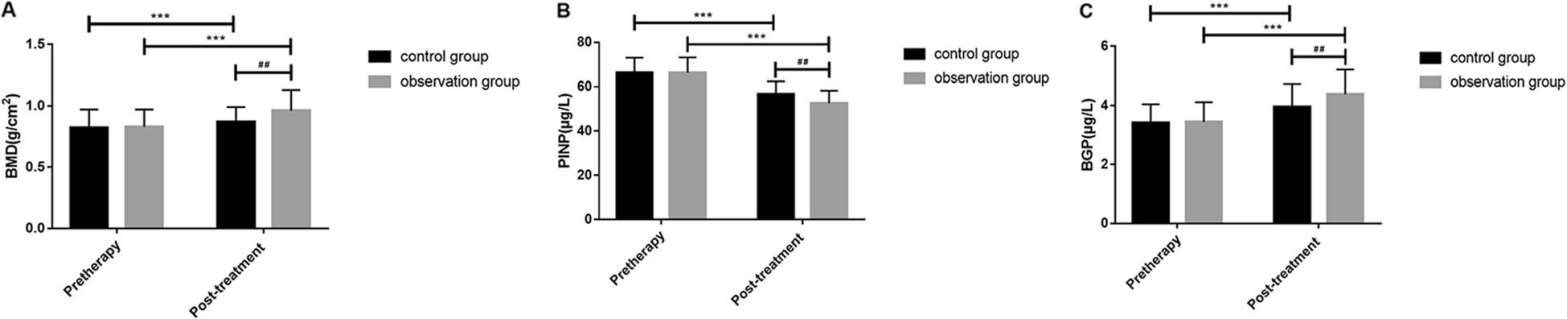
Pre and posttreatment BMD (**a**), PINP (**b**) and BGP (**c**) levels. ^***^There was a difference in a group before and after treatment (*P* < 0.001). ^##^There was a difference between two groups after treatment (*P* < 0.01). BMD, bone mineral density; PINP, procollagen type I N-terminal propeptide; BGP, bone gla protein

**Fig. 3:**
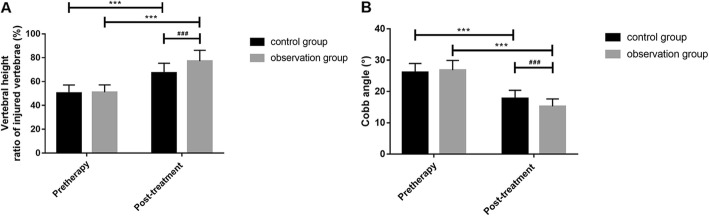
Pre and posttreatment vertebral height ratios of injured vertebrae (**a**) and Cobb angle (**b**). ^***^There was a difference in a group before and after treatment (*P* < 0.001). ^###^There was a difference between two groups after treatment (*P* < 0.001)

**Fig. 4:**
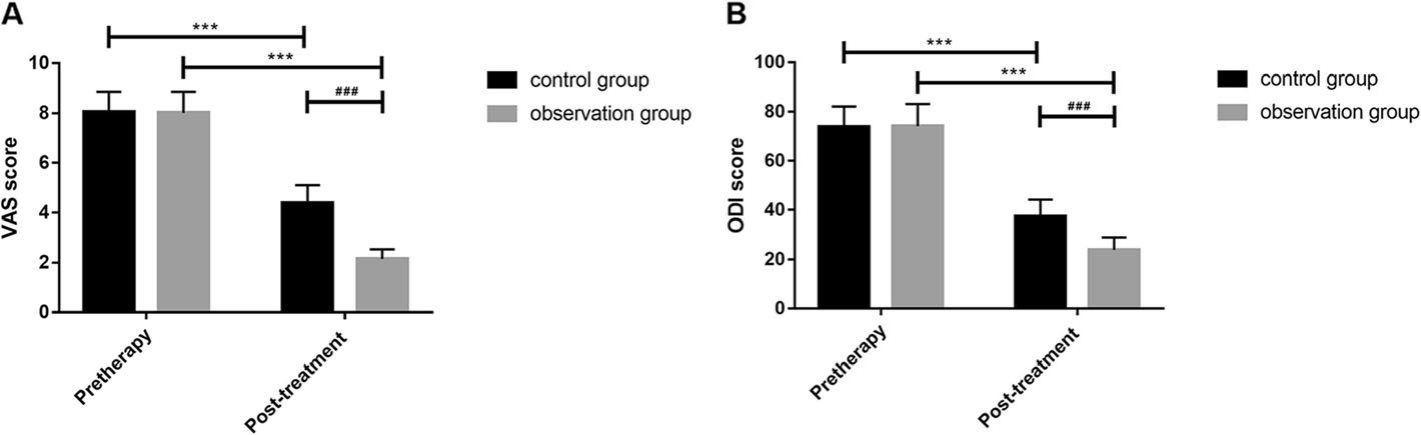
Pre and posttreatment VAS (**a**) and ODI (**b**) scores. **a** There was no significant difference in VAS scores before treatment between two groups (*P* > 0.05); VAS score in the observation group after treatment was lower than that in the control group (*P* < 0.01); there was significant difference in a group before and after treatment (*P* < 0.001). **b** There was no significant difference in ODI scores before treatment between two groups (*P* > 0.05); ODI score in the observation group after treatment was lower than that in the control group (*P* < 0.01); there was significant difference in a group before and after treatment (*P* < 0.001). ^***^There was a difference in a group before and after treatment (*P* < 0.001). ^###^There was a difference between two groups after treatment (*P* < 0.001). VAS, visual analogue scale/score; ODI, Oswestry disability index

The detection found that there were no significant differences in TNF-α and IL-6 levels in serum, BMD, PINP, and BGP levels; vertebral height ratio of injured vertebrae; and Cobb angle as well as VAS and ODI scores before treatment between the two groups (all *P* > 0.05). TNF-α and IL-6 levels in serum, PINP level, Cobb angle as well as VAS and ODI scores after a 6-month treatment were significantly reduced in both groups compared with those before treatment, while BMD and BGP levels and vertebral height ratio of injured vertebrae were significantly increased (all *P* < 0.001). There were no differences in difference values of pre and posttreatment TNF-α and IL-6 levels between the two groups (all *P* > 0.05), but changes in difference values of BMD, PINP, and BGP levels; vertebral height ratio of injured vertebrae; and Cobb angle as well as VAS and ODI scores during the 6-month treatment were significantly lower in the control group than in the observation group (all *P* < 0.01, Tables 2, 3, 4, and 5, Figs. 1, 2, 3, and 4).

**Table 2:** Difference values of pre and posttreatment tumor necrosis factor-α and interleukin-6 levels

	Control group (*n* = 52)	Observation group (*n* = 60)	*t*	*P*
TNF-α (pg/mL)	12.71 ± 5.82	12.79 ± 3.02	0.093	0.926
IL-6 (pg/mL)	11.27 ± 1.54	11.44 ± 0.94	0.547	0.586

*TNF-α* tumor necrosis factor-α, *IL-6* interleukin-6

**Table 3:** Difference values of pre and posttreatment bone mineral density, procollagen type I N-terminal propeptide and bone gla protein levels

	Control group (*n* = 52)	Observation group (*n* = 60)	*t*	*P*
BMD (g/cm^2^)	0.08 ± 0.03	1.34 ± 0.04	186.211	< 0.001
PINP (μg/L)	7.43 ± 1.40	14.24 ± 2.59	16.931	< 0.001
BGP (μg/L)	0.37 ± 0.23	0.83 ± 0.12	13.520	< 0.001

*BMD* bone mineral density, *PINP* procollagen type I N-terminal propeptide, *BGP* bone gla protein

**Table 4:** Difference values of pre and posttreatment vertebral height ratios of injured vertebrae and cobb angle

	Control group (*n* = 52)	Observation group (*n* = 60)	*t*	*P*
Vertebral height ratio of injured vertebrae (%)	17.62 ± 9.79	27.97 ± 11.26	5.152	< 0.001
Cobb angle (°)	7.30 ± 3.12	11.72 ± 4.20	6.240	< 0.001

**Table 5:** Difference values of pre and posttreatment visual analogue scale/score and Oswestry disability index scores

	Control group (*n* = 52)	Observation group (*n* = 60)	*t*	*P*
VAS score	3.63 ± 1.24	6.05 ± 1.20	10.481	< 0.001
ODI score	36.17 ± 10.53	51.15 ± 8.69	8.250	< 0.001

*VAS* visual analogue scale/score, *ODI* Oswestry disability index

### Comparison of clinical efficacy

**Fig. 5:**
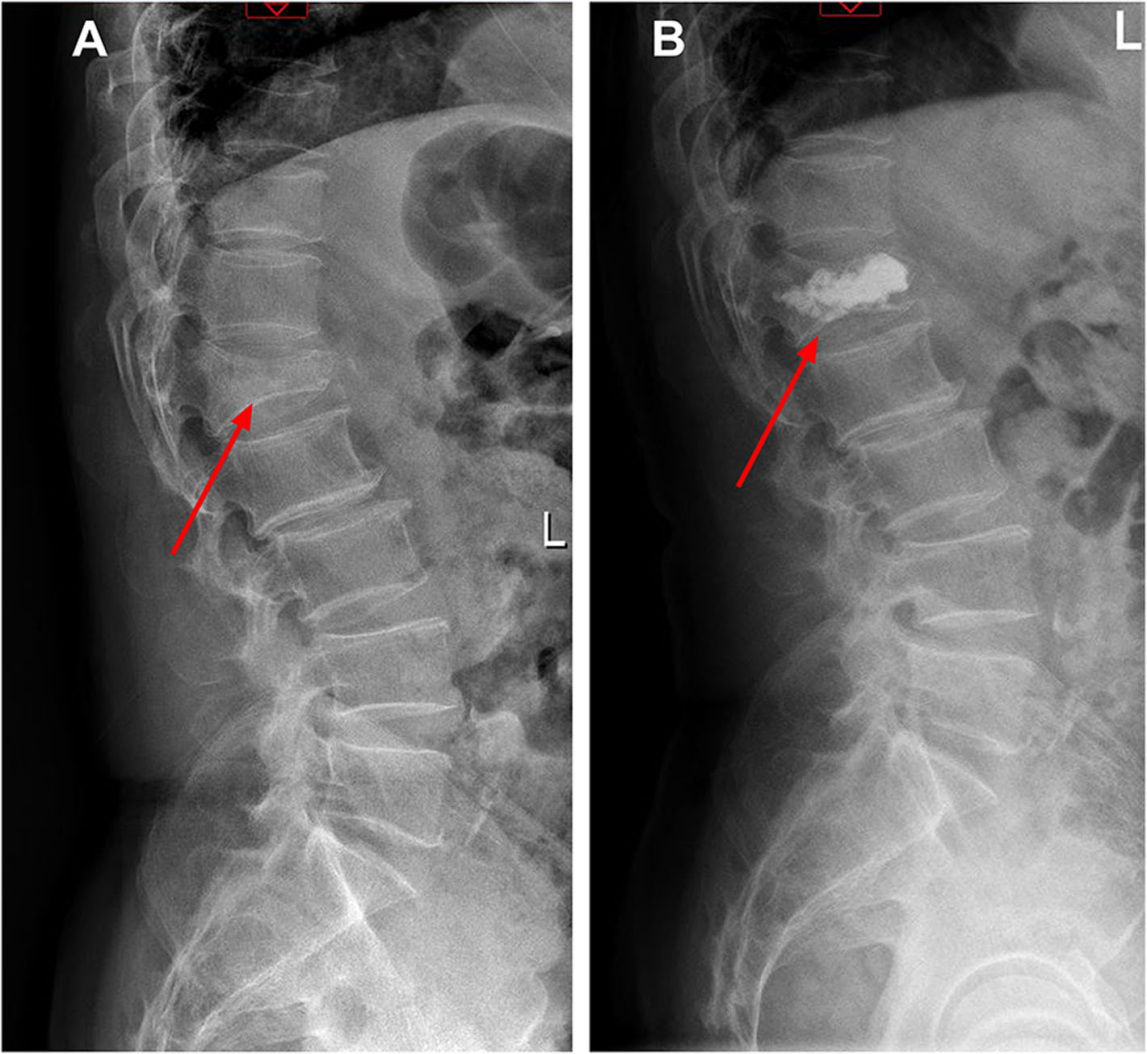
The imageological feature of a patient pre and postoperation. **a** The imageological feature preoperation. The red arrow marks the site of compression fracture. **b** The imageological feature postoperation. The red arrow marks the recovery of spinal growth post operation. The white material was bone cement that filled in.

Evaluation on clinical efficacy after treatment showed that there were 32 patients with excellent efficacy, 26 patients with general efficacy, 2 patients with failed efficacy in the observation group, while 18 patients with excellent efficacy, 28 patients with general efficacy, and 6 patients with failed efficacy in the control group. The rank sum test showed a significant difference in clinical efficacy between the two groups (*Z* = −2.233, *P* = 0.026). The imageological feature of a patient showing pre and postoperative changes was shown in Fig. 5.

### Analysis of risk factors that affecting clinical efficacy

According to clinical efficacy after treatment, patients with excellent efficacy were divided into excellent efficacy group (*n* = 50), and patients with general efficacy and failed efficacy were divided into general efficacy group (*n* = 62). The results revealed that there were differences in the use of zoledronic acid, vertebral height ratios of injured vertebrae, Cobb angle, and ODI score (all *P* < 0.05, Table 6).

**Table 6:** Univariate analysis of risk factors affecting clinical efficacy

Factor	Excellent efficacy group (*n* = 50)	General efficacy group (*n* = 62)	*t*/χ^2^/*Z*	*P*
Age (years)	51.5 ± 3.1	51.1 ± 3.4	0.629	0.531
BMI (kg/m^2^)	23.01 ± 1.62	23.22 ± 1.92	0.618	0.538
Marital status			0.615	0.735
Married	40 (80.00)	53 (85.48)		
Unmarried	3 (6.00)	3 (4.84)		
Divorced	7 (14.00)	6 (9.68)		
Past medical history				
Hypertension	15 (30.00)	21 (33.87)	0.190	0.436
Diabetes	10 (20.00)	22 (35.48)	3.252	0.071
Hyperlipidemia	12 (24.00)	18 (29.03)	0.357	0.550
Coronary heart disease	5 (10.00)	7 (11.29)	0.048	0.826
Place of residence			0.047	0.828
City	42 (84.00)	53 (85.48)		
Rural area	8 (16.00)	9 (14.52)		
Degree of education			0.155	0.734
≥ Senior high school	34 (68.00)	44 (70.97)		
< Senior high school	16 (32.00)	18 (29.03)		
Causes			0.303	0.860
Falling down	35 (70.00)	46 (74.19)		
Sprain	10 (20.00)	10 (16.13)		
Other	5 (10.00)	6 (9.68)		
Site of injury vertebrae			−1.142	0.254
T7	4 (6.56)	6 (7.23)		
T8	0 (0.00)	2 (2.41)		
T9	2 (3.28)	3 (3.61)		
T10	4 (6.56)	5 (6.02)		
T11	4 (6.56)	7 (8.43)		
T12	11 (18.03)	20 (24.10)		
L1	12 (19.67)	14 (16.87)		
L2	8 (13.11)	10 (12.05)		
L3	8 (13.11)	7 (8.43)		
L4	6 (9.84)	6 (7.23)		
L5	2 (3.28)	3 (3.61)		
Use of zoledronic acid			3.949	0.047
Used	32 (64.00)	28 (45.16)		
Unused	18 (36.00)	34 (54.84)		
Fracture acuteness				
Acute	30 (60.00)	36 (58.06)		
Subacute	20 (40.00)	26 (41.94)		
Peripheral vertebrae wall damage			2.637	0.104
Damaged	8 (16.00)	18 (29.03)		
Undamaged	42 (84.00)	44 (70.97)		
Bone cement dosage (mL)	5.08 ± 1.43	5.19 ± 1.01	0.479	0.633
TNF-α (pg/mL)	5.86 ± 1.81	6.08 ± 2.06	0.595	0.553
IL-6 (pg/mL)	11.03 ± 3.14	11.21 ± 3.40	0.289	0.773
BMD (g/cm^2^)	0.95 ± 0.21	0.92 ± 0.14	0.908	0.366
PINP (μg/L)	55.93 ± 5.82	55.18 ± 5.51	0.701	0.485
BGP (μg/L)	4.16 ± 0.73	4.13 ± 0.84	0.200	0.842
Vertebral height ratios of injured vertebrae (%)	75.02 ± 10.63	69.29 ± 9.19	6.822	< 0.001
Cobb angle (°)	15.98 ± 2.15	17.55 ± 3.54	2.757	0.007
VAS score	3.04 ± 1.65	2.80 ± 1.25	0.879	0.381
ODI score	26.52 ± 10.15	38.55 ± 7.30	7.322	< 0.001

*BMI* body mass index, *TNF-α* tumor necrosis factor-α, *IL-6* Interleukin-6, *BMD* bone mineral density, *PINP* procollagen type I N-terminal propeptide, *BGP* bone gla protein, *VAS* visual analogue scale/score, *ODI* Oswestry disability index

### Multivariate analysis of risk factors affecting clinical efficacy

Multivariate logistic regression analysis of factors having differences by univariate analysis and age found that Cobb angle was not a risk factor affecting clinical efficacy, and the use of zoledronic acid (odds ratio (OR) = 4.600, 95% confidence interval (CI) 1.483~14.266), vertebral height ratio of injured vertebrae (OR = 0.916, 95% CI 0.863~0.971), and ODI (OR = 1.224, 95% CI 1.131~1.325) were independent risk factors affecting clinical efficacy (Tables 7 and 8).

**Table 7:** Assignment

Factor	Assignment
Use of zoledronic acid	Used = 0, unused = 1
Vertebral height ratio of injured vertebrae	A continuous variable, analyzed using the original value
Cobb angle	A continuous variable, analyzed using the original value
ODI	A continuous variable, analyzed using the original value
Clinical efficacy	Excellent efficacy group = 0, general efficacy group = 1

*ODI* Oswestry disability index

**Table 8:** Multivariate logistic regression analysis

Factor	B	S.E.	Wals	Sig.	Exp (B)	EXP(B) 95% CI	
						Lower limit	Higher limit
Use of zoledronic acid	1.526	0.577	6.983	0.008	4.600	1.483	14.266
Vertebral height ratio of injured vertebrae	-0.088	0.030	8.739	0.003	0.916	0.863	0.971
Cobb angle	0.038	0.091	0.171	0.680	1.038	0.869	1.241
ODI	0.202	0.040	25.081	0.000	1.224	1.131	1.325

*ODI* Oswestry disability index

### Postoperative bone cement leakage

There were 15 patients suffering from postoperative bone cement leakage in the control group and 10 patients suffering from postoperative bone cement leakage in the observation group. There was no significant difference in the incidence of postoperative bone cement leakage between the two groups (*χ*^2^ = 2.383, *P* = 0.123).

### Univariate analysis of postoperative bone cement leakage

According to postoperative bone cement leakage, patients were divided into leakage group (*n* = 25) and no leakage group (*n* = 87). The data of age, BMI, marital status, past medical history, place of residence, degree of education, causes, site of injured vertebrae, use of zoledronic acid, fracture acuteness, peripheral vertebrae wall damage, bone cement dosage, TNF-α, IL-6, BMD, PINP, BGP, vertebral height ratios of injured vertebrae, Cobb angle, VAS score, and ODI score were analyzed by univariate analysis. The results revealed that there were differences in peripheral vertebrae wall damage and bone cement dosage (both *P* < 0.05, Table 9).

**Table 9:** Univariate analysis of postoperative bone cement leakage (*n*, %)

Factor	Leakage group (*n* = 25)	No leakage group (*n* = 87)	*t*/χ^2^/*Z*	*P*
Age (years)	51.4 ± 3.4	51.0 ± 3.2	0.591	0.556
BMI (kg/m^2^)	23.40 ± 1.80	23.29 ± 1.87	0.316	0.753
Marital status			0.460	0.795
Married	20 (80.00)	73 (83.91)		
Unmarried	2 (8.00)	4 (4.60)		
Divorced	3 (12.00)	10 (11.49)		
Past medical history				
Hypertension	7 (28.00)	29 (33.33)	0.253	0.615
Diabetes	6 (24.00)	26 (29.89)	0.330	0.566
Hyperlipidemia	4 (16.00)	26 (29.89)	1.909	0.167
Coronary heart disease	3 (12.00)	10 (11.49)	0.005	0.945
Place of residence			3.395	0.065
City	18 (72.00)	76 (87.36)		
Rural area	7 (28.00)	11 (12.64)		
Degree of education			0.485	0.486
≥ Senior high school	16 (64.00)	62 (71.26)		
< Senior high school	9 (36.00)	25 (28.74)		
Causes			0.270	0.874
Falling down	15 (6.00)	51 (58.62)		
Sprain	7 (28.00)	28 (32.18)		
Other	3 (12.00)	8 (9.20)		
Site of injured vertebrae			−0.065	0.948
T7	2 (7.41)	8 (6.84)		
T8	0 (0.00)	2 (1.71)		
T9	1 (3.70)	4 (3.42)		
T10	2 (7.41)	7 (5.89)		
T11	3 (11.11)	8 (6.84)		
T12	5 (18.52)	26 (22.22)		
L1	4 (14.81)	22 (18.80)		
L2	3 (11.11)	15 (12.82)		
L3	3 (11.11)	12 (10.26)		
L4	3 (11.11)	9 (7.69)		
L5	1 (3.70)	4 (3.42)		
Use of zoledronic acid			0.535	0.465
Used	10 (15.00)	42 (48.28)		
Unused	15 (85.00)	45 (51.72)		
Fracture acuteness			0.015	0.902
Acute	15 (60.00)	51 (58.62)		
Subacute	10 (40.00)	36 (41.38)		
Peripheral vertebrae wall damage			11.092	0.001
Damaged	12 (48.00)	14 (16.09)		
Undamaged	13 (52.00)	73 (83.91)		
Bone cement dosage (mL)	5.58 ± 1.53	4.20 ± 0.91	5.889	< 0.001
TNF-α (pg/mL)	5.76 ± 1.91	6.12 ± 2.76	0.790	0.430
IL-6 (pg/mL)	10.73 ± 3.04	11.16 ± 3.52	0.686	0.494
BMD (g/cm^2^)	0.92 ± 0.17	0.91 ± 0.15	0.330	0.742
PINP (μg/L)	54.73 ± 6.32	54.43 ± 6.15	0.254	0.800
BGP (μg/L)	4.16 ± 0.73	4.13 ± 0.84	0.842	0.200
Vertebral height ratios of injured vertebrae (%)	74.61 ± 11.40	73.33 ± 8.90	0.666	0.507
Cobb angle (°)	16.98 ± 2.84	16.55 ± 3.04	0.443	0.770
VAS score	3.04 ± 1.65	2.60 ± 1.30	1.577	0.118
ODI score	29.48 ± 10.06	30.26 ± 9.50	0.422	0.674

*BMI* body mass index, *TNF-α* tumor necrosis factor-α, *IL-6* Interleukin-6, *BMD* bone mineral density, *PINP* procollagen type I N-terminal propeptide, *BGP* bone gla protein, *VAS* visual analogue scale/score, *ODI* Oswestry disability index

### Multivariate logistic regression analysis of postoperative bone cement leakage

Multivariate logistic regression analysis of factors that have differences by univariate analysis and age found that age was not a risk factor causing postoperative bone cement leakage (*P* > 0.05), and bone cement dosage (OR = 2.845, 95% CI 2.164~3.741), and peripheral vertebrae wall damage (OR = 3.388, 95% CI 1.966~5.840) were independent risk factors causing postoperative bone cement leakage (Tables 10 and 11).

**Table 10:** Assignment

Factor	Assignment
Age (years)	< 50 years = 0, ≥ 50 years = 1
Bone cement dosage	A continuous variable, analyzed using the original value
Peripheral vertebrae wall damage	Damaged = 0, undamaged = 1

**Table 11:** Multivariate logistic regression analysis

	*β*	S.E.	Wals	Sig.	OR	95% CI
Age (years)	−0.425	0.276	2.380	0.123	0.654	0.381~1.122
Bone cement dosage	1.046	0.140	56.074	0.000	2.845	2.164~3.741
Peripheral vertebrae wall damage	1.220	0.278	19.304	0.000	3.388	1.966~5.840

*OR* odds ratio, *CI*, confidence interval

## Discussion

TNF-α level is increased in the body suffering from malignant tumors, cardiovascular and cerebrovascular diseases, chronic inflammatory diseases, and fractures [16]. IL-6, as a trigger for inflammation in the body, is an important clinical indicator of the degree of inflammation. IL-6 level in the serum is increased significantly after the fracture, and gradually decreased with the improvement of the inflammatory response [17]. The detection on TNF-α and IL-6 levels before and after treatment suggested that the inflammatory response was significantly improved after PKP treatment, and the combination of zoledronic acid and PKP did not affect the inflammatory response. In this study, we first reported the effect of PKP combined with zoledronic acid on TNF-α and IL-6 levels in serum of OVCF patients.

Bone metabolism is an important indicator of postoperative prognosis in patients with OVCF. BMD, an important indicator of bone strength, is the most effective predictor of fracture risk [18]. PINP is a product of type I procollagen by enzyme digestion, and its expression can reflect the synthesis rate of type I procollagen and the condition of bone turnover [19]. BGP is a protein secreted and synthesized by chondrocytes and osteoblasts, and the change in its expression directly reflects the activity of osteoblasts and the rate of bone turnover [20]. The observation on bone metabolism markers found that there was no difference in the expression of BMD, PINP, and BGP before treatment between the two groups. BMD, PINP, and BGP after treatment were significantly improved compared with those before treatment, and changes in difference values of BMD, PINP, and BGP during treatment were significantly higher in the observation group than in the control group. It indicated that zoledronic acid can improve the progression of bone metabolism in patients; zoledronic acid with a unique dinitrogen imidazole heterocyclic structure has strong binding ability to the bone surface, prevents osteoporosis by adjusting osteoblasts and osteoclasts, and has a low stimulation on the digestive tract by intravenous administration [21].

In this study, changes in difference values of vertebral height ratio of injured vertebrae and Cobb angle during treatment were significantly lower in the control group than in the observation group, indicating that PKP combined with zoledronic acid promoted the increase of vertebral height ratio of injured vertebrae, and the decrease of Cobb angle, improving the patient's condition. Evaluation of the efficacy showed that the clinical efficacy in the observation group using zoledronic acid was significantly better than that in the control group. A further analysis of risk factors affecting the clinical efficacy revealed that the use of zoledronic acid, vertebral height ratio of injured vertebrae, and ODI are independent risk factors affecting the clinical efficacy on patients.

As the most commonly used pain score in clinical practice, VAS score has the characteristics of simple operation and convenience and can directly reflect the pain degree of patients [22]. ODI score is a scoring standard used by clinicians to assess the degree of lower extremity disability; the higher the score, the more severe the lower extremity disability [23]. In this study, VAS score and ODI score after treatment were significantly improved compared with those before treatment, and the improvement of scores in the observation group was significantly better than that in the control group. We speculated that single PKP in the control group did not apparently improve osteoporosis while zoledronic acid and PKP in the observation group improved osteoporosis, causing better VAS score and ODI score.

Bone cement leakage has always been an important shortcoming of vertebroplasty. Although bone cement leakage has been effectively improved by PKP compared with PVP, it is still not completely avoided [24]. In this study, 25 of 112 OVCF patients who were treated with PKP had bone cement leakage. Univariate analysis of clinical data showed that peripheral vertebrae wall damage and bone cement dosage were risk factors causing bone cement leakage; multivariate logistic regression analysis showed that peripheral vertebrae wall damage and bone cement dosage were independent risk factors causing bone cement leakage. An analysis of 108 OVCF patients who were treated with PVP or PKP by Zheng et al. found that bone cement dosage is an independent risk factor causing bone cement leakage, which is consistent with the results in our study [25]. The study of Sun et al. showed that PKP is safe and reliable for the treatment of peripheral vertebrae wall damaged osteoporotic thoracolumbar vertebral fractures, only 10 vertebrae have bone cement leakage, and peripheral vertebrae wall damage is not an absolute contraindication [26]. However, in this study, we found that peripheral vertebrae wall damage was a risk factor causing bone cement leakage. It indicated that it is necessary to strictly grasp the patient's surgical indications before surgery and repeatedly study the patient's image diagnosis reports in order to determine an effective and reliable treatment regimen, reducing the occurrence of bone cement leakage.

However, this study still has certain shortcomings. Firstly, we did not conduct long-term follow-up visit to the patients in this study, and did not evaluate the long-term efficacy of the treatment. Secondly, the number of samples was small, especially in the analysis of risk factors, and we only collected patients aged 45–55 years without patients at other ages (55 years above). Therefore, we will increase the research time, the number of patients, and the sample size of elderly female patients in future research to verify the results of this study.

## Conclusions

In summary, peripheral vertebrae wall damage and bone cement dosage are independent risk factors causing bone cement leakage in patients with OVCF treated with PKP. PKP combined with zoledronic acid can effectively alleviate the condition of OVCF postmenopausal women, reduce the occurrence of inflammatory response, and improve the quality of life and pain of patients, and is worthy of clinical popularization.

## Data Availability

The datasets used and/or analyzed during the current study are available from the corresponding author on reasonable request.

## Abbreviations

OVCF: Osteoporotic vertebral compression fracture
PKP: Percutaneous kyphoplasty
PVP: Percutaneous vertebroplasty
IL-6: Interleukin-6
TNF-α: Tumor necrosis factor-α
PINP: Serum procollagen type I N-terminal propeptide
BGP: Serum bone gla protein
BMD: Bone mineral density
VAS: Visual analogue scale/score
ODI: Oswestry disability index
BMI: Body mass index
OR: Odds ratio
CI: Confidence interval
